# Using entrustable professional activities to better prepare students for their postgraduate medical training: A medical student’s perspective

**DOI:** 10.1007/s40037-022-00731-x

**Published:** 2022-11-28

**Authors:** Sarah E. Kuehl, Jennifer O. Spicer

**Affiliations:** 1 Emory University School of Medicine and Goizueta Business School, Atlanta, GA USA; 2 J. Willis Hurst Internal Medicine Residency Program, Division of Infectious Diseases, Department of Medicine, Emory University School of Medicine, Atlanta, GA USA

**Keywords:** Entrustable professional activity (EPA), Medical student, Competency-based medical education, Undergraduate medical education

## Abstract

**The problem:**

Medical students graduate underprepared for postgraduate medical training despite years of classroom and clinical training. In this article, a medical student shares her personal perspectives on three factors contributing to this problem in undergraduate medical education: students’ peripheral roles in the clinical environment impede learning, students receive inadequate feedback, and assessments do not measure desired learning outcomes.

**A solution:**

The authors describe how using entrustable professional activities (EPAs) could address these issues and promote students’ clinical engagement by clarifying their roles, providing them with frequent and actionable feedback, and aligning their assessments with authentic work. These factors combined with grading schemes rewarding improvement could contribute to a growth mindset that reprioritizes clinical skill acquisition. The authors explore how medical schools have begun implementing the EPA framework, highlight insights from these efforts, and describe barriers that must be addressed.

**The future:**

Incorporating EPAs into medical school curricula could better prepare students for postgraduate training while also alleviating issues that contribute to student burnout by defining students’ roles, improving feedback, and aligning assessments with desired learning outcomes.

**Supplementary Information:**

The online version of this article (10.1007/s40037-022-00731-x) contains supplementary material, which is available to authorized users.

## Introduction

Medical students graduate underprepared for postgraduate medical training despite years of intense classroom and clinical training [1]. New graduates feel that they lack all the necessary skills for their new roles [2–5], and their supervisors express similar concerns [3, 6, 7]. To address this issue, there has been an increased focus on how to better prepare students for their transition to postgraduate training. Many schools have concentrated on developing new curricula in the final year of medical school to aid with the transition, which has helped [8–10]; however, from my perspective as a current medical student, there are additional changes that need to be made.

In this paper, I outline three factors contributing to this issue: peripheral student roles, lack of formative feedback, and misaligned learner assessments that underemphasize improvement. Then, I explain how the incorporation of entrustable professional activities (EPAs) into medical school curricula provides a potential solution. Finally, I provide examples of how EPAs have already been implemented in medical school curricula and lessons learned from those interventions.

## Three factors contributing to students being underprepared for postgraduate training

### Peripheral student roles reduce learning opportunities

Students are often relegated to the periphery of the clinical team with limited opportunities for active engagement in patient care [11]. There are likely several reasons for this phenomenon. First, rotations can be brief, and time with specific faculty or junior doctors can be even shorter, leading to insufficient time for supervisors to determine students’ abilities and appropriate levels of autonomy. Second, students’ uncertainty regarding their responsibilities can prevent them from pursuing active roles in patient care activities. Finally, active student engagement has decreased over time due to the introduction of the electronic health record [12] and an increased culture of direct supervision. Thus, it is common for medical students to feel like they are “shadowing” rather than training on rotations.

Although some would suggest students should advocate for more clinical engagement, a few barriers exist. Students’ concerns about performance, grades, and hierarchy interfere with appropriate advocacy [11]. Students might worry that asking to see patients on their own inconveniences faculty or appears disrespectful if they have not had time to build a relationship [11]. Additionally, students lacking confidence in underdeveloped areas may not want to demonstrate skills needing improvement due to fear of poor evaluations or concerns of bothering patients. Thus, placing the impetus solely on learners to ask for more clinical involvement is problematic.

The tendency toward peripheral student roles combined with students’ hesitancy to advocate for themselves impedes students’ clinical development. Active involvement is essential to learn clinical skills. If students do not have the opportunity to practice their skills, they have difficulty learning clinical medicine and miss opportunities to receive meaningful feedback from supervisors.

### Current feedback results in limited student improvement

Feedback occurs infrequently and often lacks concrete suggestions for improvement; phrases such as “read more” are frequently written in evaluations, with limited comments regarding specific strengths and areas for improvement [13, 14]. Students may only receive feedback in their formal rotation evaluations due to supervisors’ time constraints or students’ apprehension with asking. Without meaningful, frequent feedback, clinical learning often occurs through observation followed by personal trial and error rather than guided instruction. Students’ mistakes may go unnoticed and best practices may not be adequately developed.

Infrequent opportunities to engage in observed clinical duties with feedback contributes to a performance mindset that is prevalent among learners. In a performance or fixed mindset, students hide areas in need of development in order to appear competent, thus inhibiting supervisors from helping them improve. In medical education, the goal is to promote a growth mindset where learners work to improve weaknesses through calculated efforts and coaching [15].

Students, however, believe improvement has little influence on evaluation scores [13]. Instead, students view observations as supervisor assessments rather than as opportunities for growth [16]. Since securing postgraduate training positions has become increasingly competitive, many students feel they must outperform their peers. Thus, students strive to receive top evaluations by hiding weaknesses. For example, some medical students receive advice from peers to only ask questions to which they know the answer in order to appear competent if the question is “fired back”. The combination of infrequent, inadequate feedback and promotion of a performance mindset among students inhibits the development of clinical skills.

### Assessments do not measure desired outcomes

There are two overarching issues with many assessment systems in undergraduate medical education. First, they incentivize students to prioritize learning medical knowledge over clinical skills [7, 17]. Because medical students perceive clinical evaluations to be highly subjective and lacking in variability [13], my experience is that students feel standardized multiple-choice examinations are the only portion of their grade under their control that will differentiate them from their peers. Therefore, students spend more time studying for those exams than engaging in clinical care since those exams will heavily influence their grades and, subsequently, their competitiveness for selection into postgraduate training programs. Thus, students struggle to focus on their true interest, clinical learning, given the pressure to receive top grades.

Second, summative clinical assessments often do not accurately measure students’ clinical skills [13, 18, 19]. In a survey of nearly 350 students from one school, over half reported never being observed by a faculty member or junior doctor while they conducted a physical exam [18]. Instead, this skill is commonly evaluated indirectly through oral presentations in the clinical setting. Moreover, the subjectivity of evaluations is supported by one survey of over 700 students across 6 U.S. schools—when asked about perceived importance of various factors toward rotation grades, “being liked”, “particular attendings”, or “particular junior doctors you work with” were deemed most important [13]. Even more worrisome is how subjectivity may be disproportionately impacting students from backgrounds underrepresented in medicine [19].

Because of these factors, clinical skills are inaccurately evaluated. Since grading structures are known to influence student priorities [17, 20] students may prioritize learning objectively evaluated medical content over building clinical skills. Because success on standardized medical knowledge exams does not necessarily predict success as a junior doctor [21], the assessment structure within medical schools likely contributes to students being underprepared for postgraduate training.

## How EPA frameworks offer solutions for medical schools

Over the last few decades, medical education has been shifting to a competency-based medical education model, which is an outcomes-based educational model that relies upon formative, workplace-based assessments rather than the traditional summative evaluation approach [22, 23]. Entrustable professional activities (EPAs), which are discrete units of professional work, were developed to put competencies into context and to help evaluators focus on specific professional activities [24, 25]. Initially EPAs were primarily used in postgraduate training; however, EPAs are increasingly being used in undergraduate medical education as well [26, 27]. Creating a standardized list of EPAs and integrating them into medical school curricula directly addresses variance in first-year trainee skills by clearly delineating the skills medical students are expected to have upon graduation. This guides each medical school’s curricular content to ensure these minimum expectations are met. Additionally, if required for graduation, they ensure that all students gain those skills prior to graduating medical school and entering postgraduate training. If students’ abilities are deemed insufficient, they must improve until they meet the designated threshold.

A report outlining recommendations to improve the undergraduate to graduate transition stated the need to define and implement a set of educational outcomes [28]. To date, three countries have developed a national list of EPAs to describe activities that students should be able to complete unsupervised upon graduation [29–31]. In the US, these were developed by the Association of American Medical Colleges (AAMC) and contain 13 Core EPAs. In 2014, the AAMC recruited ten medical schools in the United States to participate in the Core EPAs Pilot, an initiative aimed at implementing their framework [32]. Medical students from those institutions recently expressed their perspectives about this process and notably did not recommend making EPAs a component of graduation requirements at this time [33]. Their perspective is understandable given the high stakes behind these EPA assessments and their current uncertain reliability and validity; however, schools should continue to strive to address these issues as one of the key advantages of using an EPA framework in this way lies in its ability to standardize the skills of graduating students. Until they become a requirement for graduation or course completion, that opportunity is lost. In addition to preparing students for postgraduate training, the EPAs could provide solutions to the aforementioned problems in undergraduate medical education by promoting student clinical engagement, improving feedback, and aligning assessments with authentic work (see the figure in the Electronic Supplementary Material).

### EPAs promote increased student clinical engagement

EPAs can promote clinical engagement by clarifying students’ roles. For example, the AAMC Core EPAs were developed to serve as a national standard of skills medical students are expected to obtain, yet there is evidence some of these skills are not currently viewed as students’ roles within the US. In one study that examined written evaluations of third-year medical students, few mentioned diagnostic tests, patient handovers, recognition of urgent patient care, patient safety, orders/prescriptions, or informed consent—all skills included in the AAMC Core EPAs [34]. In countries that create national or international lists of EPAs, student responsibilities are being clarified, which the literature argues promotes increased involvement in clinical activities [35, 36]. Faculty and administrators could use EPA frameworks to identify gaps in their curriculum and incorporate new opportunities to perform EPAs not currently practiced by learners. Additionally, medical students could use EPA frameworks to guide their own learning and advocate for more clinical involvement, as they did in a program piloting the use of EPAs [37].

### Using EPAs can improve clinical feedback

EPAs can improve the content, reliability, and quantity of feedback. EPAs can improve feedback content by encouraging evaluators to focus on tasks deemed most important to learn. They can improve reliability by clearly defining expected behaviors of learners at various performance levels. For example, the AAMC has developed vignettes describing students at different learning levels for each Core EPA to improve feedback reliability [38]. This creates clear shared expectations for both students and faculty by identifying skills and describing what mastery entails. This shifts evaluations to criterion-based rather than norm-based assessments, though faculty development to implement these scales would be needed. Finally, they can increase the quantity of feedback. The incorporation of EPAs has been proven to increase the quantity of direct observation and feedback [39, 40]. For example, one medical school that implemented EPAs saw a 10-fold increase in the average number of observations with feedback per month when compared to the school’s traditional approach to assessment [40]. One plausible explanation for this is that the EPA framework gives evaluators a shared mental model of standardized student skills and thus facilitates assessments of students even if faculty only work with a student during a single clinic day. The results from this same medical school’s EPA assessment program also demonstrated external validity [41], demonstrating the potential for EPAs to improve both feedback quantity and reliability.

### EPAs align education with authentic work

Literature supports the claim that EPA assessments test something different from medical knowledge examinations. For example, one study of third-year medical students developed a rubric to assess students’ clinical notes, which is one of the AAMC Core EPAs; in that study they found no significant correlation between students’ scores on their clinical notes and their standardized end-of-rotation medical knowledge exams [42]. Additionally, some studies have found a negative correlation between standardized medical knowledge exams and EPA-based assessments [7, 17]. These findings support the view that prioritizing the acquisition of medical knowledge can come at the expense of gaining clinical skills.

With EPA-based curricula, student priorities can be realigned toward critical thinking and clinical skills. The medical students from the AAMC pilot program recommended that EPA-based assessments should remain strictly formative rather than being graded [33]; however, in rotations that are not pass/fail, including EPAs as a graded component is critical to ensure that students prioritize them. Changing grading structures has been shown to change student priorities [17, 20]. For example, in one study that aimed to promote clinical skills and critical thinking during the surgery rotation, a new curriculum added four graded components that prioritized clinical skills or critical thinking and de-emphasized the importance of the end-of-rotation medical knowledge exam [17]. Compared to students in the prior curriculum, students in the new curriculum performed significantly better on their Objective Structured Clinical Exam and on an exam that prioritized critical thinking but worse on their end-of-rotation medical knowledge exam. Considering the influence of grading structures, the recent advice to students on how to succeed in competency-based medical education programs that encouraged students to adopt a growth mindset should be considered with caution [43]. Given most medical students are highly motivated and want to learn, a growth mindset is more likely to be adopted if grading of rotations is changed to pass/fail or improvement and acknowledgement of weaknesses are explicitly included in EPA evaluations.

## Barriers to integration of EPAs in medical schools

Several medical schools have begun to implement EPAs into their curricula [27]. In the US, the AAMC Core EPAs pilot project is an attempt to incorporate competency-based medical education into medical schools, and the latter have been evaluating the feasibility of teaching and assessing their EPA framework and operationalizing the concept of entrustment for each as part of this process [44]. Based on their pilot, they released a paper that revealed key challenges to this effort from medical students’ perspectives [33]. Some questions included: who should drive the assessment process between students and supervisors, what feedback mechanisms are required, and what systems are needed to advise, mentor, and coach students. These questions highlight challenges in EPA implementation and mirror current problems within medical schools. Lack of trustworthy assessments and underdeveloped feedback systems have repeatedly been cited as barriers to competency-based medical education [45], yet these are the very issues that must be addressed within undergraduate medical education regardless of whether EPAs are implemented.

Many barriers identified from other papers demonstrate this same pattern: assessments of competencies are difficult due to fractured learning environments and inadequate faculty development [46], clinical environments do not readily accommodate frequent direct observation of learners [47, 48], and a competency-based medical education assessment system may be perceived as being in conflict with our current norm-based assessment models that emphasize the importance of being able to discriminate between learners [47, 49]. Longitudinal relationships are increasingly mentioned as necessary complements to competency-based medical education to address these problems [33, 37, 50]. Longitudinally integrated rotations are a potential avenue to achieve these relationships, but also require substantial work to implement. These challenges demonstrate there is significant work to be done to facilitate the spread of competency-based medical education; however, these efforts also strikingly align with what is needed to improve our current system anyway.

## Conclusion

The problem of graduates being underprepared for postgraduate training is well documented. This paper evaluated how peripheral student roles, inadequate feedback, and misaligned assessments contribute to this issue, and how EPAs could address these issues. Importantly, these problems within undergraduate medical education do not just detract from the readiness of graduates. Students know they should not be spending long hours only passively engaged and preparing for standardized medical knowledge exams that do not predict their future clinical skills. They feel the tension between the explicit goals of medical school, which emphasize spending time with patients and gaining clinical skills, versus the implicit goals of hiding their weaknesses to receive excellent evaluations and performing well on standardized medical knowledge examinations to secure a position at their desired postgraduate training program. This tension is exhausting and, by pulling students away from patients, it can slowly wear down their humanism, replacing it with cynicism and burnout.

## Supplementary Information

Figure summary of contributing factors to students graduating underprepared and how EPAs address these issues
