# Supplementary material for: Using entrustable professional activities to better prepare students for their postgraduate medical training: A medical student’s perspective
Source: Perspect Med Educ. 2022 Nov 28;11(6):359–64. doi: 10.1007/s40037-022-00731-x (PMC9743878; doi:10.1007/s40037-022-00731-x)
Supplement: Supplementary file 1 — Figure summary of contributing factors to students graduating underprepared and how EPAs address these issues [file 40037_2022_731_MOESM1_ESM.docx]

**Solutions**

**Problems in UME that contribute to underprepared graduates**

- Clarifies learners’ roles
- Tool to advocate for increased student clinical involvement
- Improves content by focusing evaluators on Core EPA tasks
- Improves reliability through vignettes and entrustability scales
- Increases feedback quantity
- Increases data used in evaluations through promotion of student clinical involvement and increased feedback
- Aligns education with authentic work
- Overall: promotes mastery learning of standardized skills and growth mindset

**Peripheral student role**

**Poor feedback**

**Misaligned assessments**

**Students graduate underprepared for residency**
